# Changes in hepatitis B virus surface antibody titer and risk of hepatitis B reactivation in HBsAg-negative/HBcAb-positive patients undergoing biologic therapy for rheumatic diseases: a prospective cohort study

**DOI:** 10.1186/s13075-018-1748-z

**Published:** 2018-11-01

**Authors:** Ya-Chih Tien, Hsu-Heng Yen, Ching-Fang Li, Mei-Ping Liu, Yin-Tzu Hsue, Ming-Hui Hung, Ying-Ming Chiu

**Affiliations:** 1 Division of Allergy, Immunology and Rheumatology, Department of Internal Medicine, Changhua Christian Hospital, 135 Nanxiao Street, Changhua City, 500-06 Taiwan; 2 Division of Gastroenterology, Department of Internal Medicine, Changhua Christian Hospital, Changhua City, Taiwan; 3 General Education Center, Chienkuo Technology University, Changhua City, Taiwan; 4 Division of Allergy, Immunology and Rheumatology, Department of Internal Medicine, Lukang Christian Hospital, LuKang, Taiwan; 5 Division of Allergy, Immunology and Rheumatology, Department of Internal Medicine, Yuanlin Christian Hospital, Changhua County, Taiwan; 6 Department of Nursing, College of Nursing, HungKuang University, Taichung, Taiwan

**Keywords:** DMARDs (biologic), Hepatitis B virus surface/core antibody (HBsAb/HBcAb), Rheumatic diseases, HBV reactivation

## Abstract

**Background:**

Our aim was to investigate dynamic changes in hepatitis B virus (HBV) surface antibody (HBsAb) titer and the associated risk of HBV reactivation and clinical course in patients with HBV surface antigen negative/core antibody positive (HBsAg^−^/HBcAb^+^) serostatus during antirheumatic therapy with biologic agents.

**Methods:**

In a prospective study from January 2013 to June 2017, we monitored the HBV serostatus of HBsAg^−^/HBcAb^+^ patients undergoing biologic therapy for rheumatic diseases. From HBsAb titers at baseline and subsequent time points, we calculated the person-years (PY) contributed by patients with different HBsAb levels: < 10 mIU/mL (negative); 10–100 mIU/mL (low); and > 100 mIU/mL (high). We analyzed the incidence of detectable HBV DNA and HBV reactivation in each group, and documented the clinical courses of patients.

**Results:**

Among 380 participants, 83 (21.8%) had baseline HBsAb < 10 mIU/mL, 156 (41.1%) HBsAb 10–100 mIU/mL, and 141 (37.1%) HBsAb > 100 mIU/mL. Total PY at study end were 169.3 PY from the HBsAb-negative group, 362.7 PY from the low-titer group, and 285.8 PY from the high-titer group. Seventeen patients had detectable HBV DNA, with respective incidence rates in negative, low- and high-titer groups of 4.7/100 PY, 2.5/100 PY, and 0/100 PY. Two HBsAb-negative patients subsequently developed HBV reactivation, an incidence of 1.2/100 PY.

**Conclusions:**

The risk of HBV reactivation varied with HBsAb titer, which changed during biologic therapy. Neither HBV DNA nor reactivation were detected in patients with HBsAb > 100 mIU/mL, whereas HBV DNA without reactivation occurred periodically in patients with HBsAb 10–100 mIU/mL; HBsAb-negative serostatus was associated with a risk of HBV reactivation.

**Electronic supplementary material:**

The online version of this article (10.1186/s13075-018-1748-z) contains supplementary material, which is available to authorized users.

## Background

Hepatitis B virus (HBV) reactivation has emerged as a major complication in patients with hepatitis B surface antigen-negative/core antibody-positive serostatus (HBsAg^−^/HBcAb^+^; which indicates a past HBV infection since resolved) who receive biologic agents to treat rheumatic diseases such as rheumatoid arthritis, ankylosing spondylitis, and psoriasis/psoriatic arthritis [1–6]. HBV reactivation incidence rates from 2% to 11% have been reported, depending on how HBV reactivation was defined and in which patient population [7]. The clinical spectrum of HBV reactivation ranges from asymptomatic to fulminant hepatitis, liver failure, and even death [8].

HBV surface antibody (HBsAb) has conventionally been thought to ward off HBV reactivation; loss of HBsAb precedes HBV DNA manifestation and HBV reactivation [8]. A large study in Japan showed recently that negative or low HBsAb titer (< 100 mIU/mL) at baseline in HBsAg^−^/HBcAb^+^ patients receiving immunosuppressive therapy for rheumatic diseases appeared to increase the likelihood of HBV reactivation [9]. On the other hand, patients with high baseline HBsAb titer (> 100 mIU/mL) preceding chemotherapy or immunosuppressive therapy had lower risk of HBV reactivation [10, 11]. Therefore, stratifying the risk of HBV reactivation according to baseline HBsAb has been proposed [5–7]. In addition, other studies showed that HBsAb titer decreased after chemotherapy [11–13] or biologic therapy [14, 15]. However, there has been no study exploring if the risk of HBV reactivation varies followed by the change of HBsAb titer during biologic therapy.

Given that the HBsAb titer changes after administering biologic agents, the risk of HBV reactivation might also vary, depending on the changing HBsAb titer over time, rather than remaining at the level associated with the baseline titer. To investigate this hitherto unexplored hypothesis, we monitored the dynamic changes in HBsAb titer during treatment for rheumatic diseases with biologic agents, and the associated risk of HBV reactivation.

## Methods

### Study participants

**Fig. 1:**
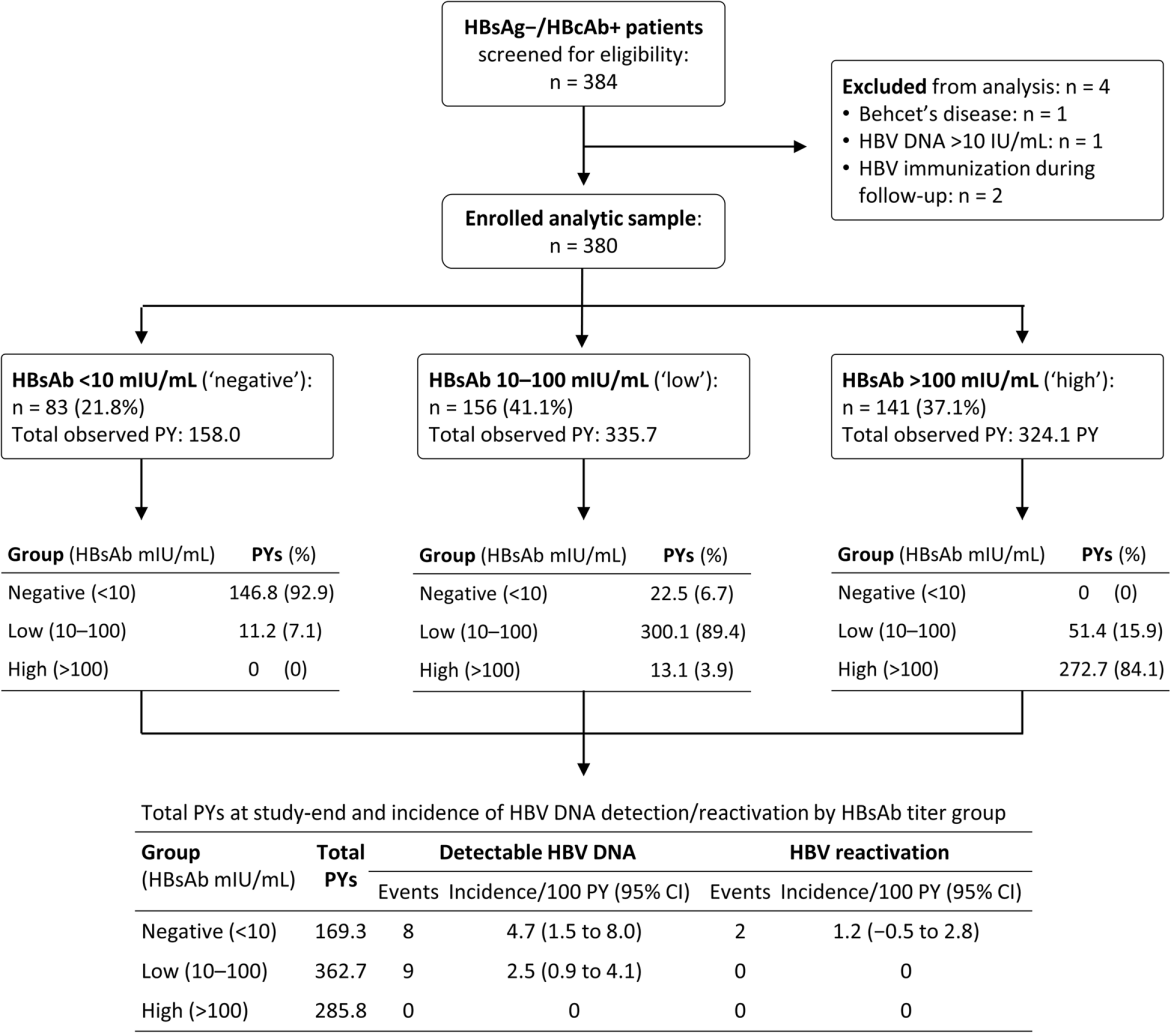
Hepatitis B virus surface antibody (HBsAb) titers, cumulative person-years (PY), and incidence rates of detectable hepatitis B virus (HBV) DNA and reactivation. CI confidence interval, HBcAb hepatitis B virus core antibody, HBsAg hepatitis B virus surface antigen

This prospective study included patients with HBsAg^−^/HBcAb^+^ serostatus who attended Changhua Christian Hospital Department of Rheumatology from January 2013 through June 2017 to receive biologic therapy (Fig. 1). Of 384 such patients, four were excluded: one with Behcet’s disease, one with baseline HBV DNA > 10 IU/mL, and two immunized against HBV during follow-up. All 380 enrolled HBsAg^−^/HBcAb^+^ patients tested negative for HBV DNA, and none had received antiviral prophylaxis. Twenty-four of these patients had hepatitis C virus co-infection. The status of autoimmune hepatitis was not screened in this cohort.

### Data collection

Blood counts, serum alanine transaminase, and HBV serostatus, including HBsAg, HBsAb, and HBV DNA, were assayed at baseline and monitored according to contemporary Taiwan guidelines [7, 16] approximately every 6 months. HBsAg, HBcAb, and HBsAb were quantified using the Architect i2000sr System chemiluminescent microparticle immunoassays (Abbott Laboratories, Abbott Park, Illinois, USA), and HBV DNA using the m2000rt RealTime™ HBV system (Abbott Laboratories), which has a detection limit of 10 IU/mL.

### Changes in HBsAb titer during follow-up

At each follow-up time point, patients were divided into groups of HBsAb titers: < 10 mIU/mL (denoted ‘negative’, or loss of antibody); 10–100 mIU/mL (denoted ‘low’); and > 100 mIU/mL (denoted ‘high’). The person-years (PY) that each patient contributed to different HBsAb titer groups during follow-up were summed.

### Primary outcomes

The primary outcomes were detectable HBV DNA, defined as viral load > 10 IU/mL (since HBV DNA < 10 IU/mL was considered negative), and HBV reactivation, defined as conversion from HBsAg^−^ to HBsAg^+^ serostatus with an absolute increase in HBV DNA > 2000 IU/mL [17, 18].

### Statistical analyses

The demographic characteristics of participants were summarized. Continuous variables are expressed as mean ± standard deviation. For the interval between each follow-up time point, patients were assigned into ‘negative’, ‘low’, and ‘high’ groups, depending on their HBsAb titers; patients could switch between groups if their HBsAb titer changed during a subsequent interval. PYs of each HBsAb group were calculated from enrolment until study end, detection of HBV DNA, or HBV reactivation, whichever occurred first. Incidence rates of detectable HBV DNA and HBV reactivation per 100 PY, with 95% confidence interval (CI), were calculated.

## Results

### Participant characteristics

Table 1 summarizes the baseline data of the 380 participants who were predominantly female, older than 50 years of age, and with rheumatoid arthritis. Etanercept and adalimumab were the most commonly used biologic agents.

**Table 1:** Baseline characteristics of patients with HBsAg^−^/HBcAb^+^ serostatus

Number of patients	380
Mean age (years)	56.3 ± 12.3
Sex (female)	242 (63.7%)
Number (%) with disease	
Rheumatoid arthritis	272 (71.6%)
Ankylosing spondylitis	69 (18.2%)
Psoriasis/psoriatic arthritis	39 (10.3%)
Mean disease duration (years)	
Rheumatoid arthritis (*n* = 156)	8.7 ± 7.1
Ankylosing spondylitis (*n* = 33)	13.3 ± 9.1
Psoriasis/psoriatic arthritis (*n* = 24)	11.5 ± 8.8
Number (%) using biologic agents	
Etanercept	124 (32.6%)
Adalimumab	106 (27.9%)
Golimumab	63 (16.6%)
Abatacept	18 (4.7%)
Tocilizumab	16 (4.2%)
Rituximab	44 (11.6%)
Ustekinumab	8 (2.1%)
Tofacitinib	1 (0.3%)
Number (%) using methotrexate	250 (65.8%)
Average dose of methotrexate (mg/week)	12.8
Number (%) using glucocorticoids	250 (65.8%)
Average dose of glucocorticoid (mg/day)	6.3
Number (%) using sulfasalazine	265 (69.7%)
Number (%) using hydroxychloroquine	216 (56.8%)
Number (%) using cyclosporine	81 (21.3%)

*HBcAb* hepatitis B virus core antibody, *HBsAg* hepatitis B virus surface antigen

### HBsAb titer changes, HBV DNA, and HBV reactivation during follow-up

Figure 1 tracks the changes of HBsAb titer and PYs contributed to the ‘negative’, ‘low’, and ‘high’ groups throughout the study. The HBsAb titer varied during follow-up; most participants remained in the same titer group as at baseline, and a proportion exhibited a declining trend in HBsAb titer, while the titer rose in a few.

Additional file 1: Figure S1 illustrates the titers of HBsAb from baseline to the end of follow-up of all patients. In the patients with HBV reactivation (two patients), the mean titer of HBsAb at baseline and the end of follow-up was 50.2 mIU/mL and 1.9 mIU/mL, while it was 185.3 mIU/mL and 153.5 mIU/mL in the patients without HBV reactivation (378 patients), respectively.

HBV DNA was detected in 17 patients: eight in the ‘negative’ group, and nine in the ‘low’ group; two of 17, both in the ‘negative’ group, had subsequent HBV reactivation. Figure 1 shows the incidence rates of detectable HBV DNA and reactivation in each group. The incidence rate of HBV reactivation is 1.2/100 PY in the ‘negative’ group, while it is zero in both the ‘low’ and ‘high’ groups.

### Association between changes in HBsAb titer and outcomes

Table 2 summarizes the HBV serostatus at baseline and when HBV DNA was detected, and the clinical course, of the 17 patients with detectable HBV DNA. In 15, none of whom received preemptive antiviral therapy, HBV DNA became undetectable spontaneously or persisted to fluctuate without HBV reactivation. Two of these 15 switched from rituximab to tocilizumab and another discontinued rituximab after HBV DNA was detected; however, the 12 others did not switch biologic therapy when HBV DNA was detected. Two (cases 16 and 17) subsequently developed HBV reactivation. HBsAb in case 16 remained < 10 mIU/mL; transient HBV DNA of 12 IU/mL during rituximab therapy later became undetectable, after which rituximab was switched to abatacept. Positive seroconversion of HBsAg with persistently elevated HBV DNA (7571 IU/mL) occurred 5 months after initiating abatacept, and HBV reactivation was diagnosed. HBsAb in case 17 was ‘low’ (93 mIU/mL) at baseline, with undetectable HBV DNA. A decline in HBsAb (63 mIU/mL) accompanied with detectable HBV DNA (90 IU/mL) was recorded 5 months after the first cycle of rituximab. A second cycle of rituximab was given; however, at the next blood test, 7 months after detecting HBV DNA, HBsAb had become ‘negative’, accompanied by HBsAg^+^ seroconversion and high HBV DNA (14,986,467 IU/mL); HBV reactivation was diagnosed.

**Table 2:** HBV serostatus and clinical course of 17 cases with detectable hepatitis B virus (HBV) DNA during biologic therapy

ID	Disease	Baseline					At HBV DNA detection						Clinical course after HBV DNA detected		
		HBV serostatus			ALT (IU/mL)	DMARDs	Months since enrolled	HBV serostatus			ALT (IU/mL)	DMARDs	Medication change on detecting HBV DNA	Preemptive antiviral therapy	Serial HBV DNA and outcome
		sAg (+/−)	sAb (mIU/mL)	DNA (IU/mL)				sAg (+/−)	sAb (mIU/mL)	DNA (IU/mL)					
1	RA	–	< 10	< 10	18	Pd, RTX	9	–	< 10	31	27	Pd, RTX	No	No	Fluctuating without reactivation
2	RA	–	< 10	< 10	20	Pd, MTX, ETA	30	–	< 10	14	27	MTX, ETA	No	No	Spontaneously became undetectable without reactivation
3	AS	–	NA	< 10	13	ADA	27	+	< 10	99	9	ADA	No	No	Fluctuating without reactivation
4	RA	–	< 10	< 10	20	Pd, MTX	27	–	< 10	17	18	Pd, MTX, RTX	RTX → TCZ	No	Spontaneously became undetectable without reactivation
5	RA	–	< 10	< 10	17	Pd, MTX, GOL	5	–	< 10	13	22	Pd, MTX, RTX	No	No	Fluctuating without reactivation
6	RA	–	< 10	< 10	30	Pd, ETA	20	–	< 10	464	17	Pd, ETA	No	No	Spontaneously became undetectable without reactivation
7	RA	–	16.7	< 10	18	Pd, MTX	36	–	< 10	14	17	Pd, MTX, RTX	No	No	Fluctuating without reactivation
8	RA	–	23.8	< 10	16	MTX, ETA	28	–	12.8	30	14	MTX, ETA	No	No	Spontaneously became undetectable without reactivation
9	RA	–	152	< 10	17	Pd, MTX, RTX	30	–	68	22	13	Pd, MTX, RTX	No	No	Spontaneously became undetectable without reactivation
10	RA	NA	110	< 10	34	Pd, RTX	7	NA	83	435	54	Pd, RTX	Hold RTX	No	No reactivation
11	RA	–	26	< 10	16	MTX, ETA	48	–	85	11	20	Pd, MTX, GOL	No	No	Spontaneously became undetectable without reactivation
12	RA	–	44	< 10	27	Pd, MTX, ABA	17	–	38	11	45	Pd, MTX, RTX	No	No	Fluctuating without reactivation
13	RA	–	NA	< 10	16	Pd, MTX	27	–	93	66	20	Pd, MTX, ADA	No	No	No reactivation
14	RA	–	47.2	< 10	26	Pd, MTX, ADA	24	–	26	35	36	Pd, MTX, RTX	RTX → TCZ	No	Spontaneously became undetectable without reactivation
15	RA	–	4	< 10	39	Pd, MTX	39	–	14	11	23	MTX, ETA	No	No	Fluctuating without reactivation
16	RA	NA	< 10	< 10	36	Pd, LEF, RTX	15	–	< 10	12	36	Pd, LEF RTX	No	No	Fluctuated to become undetectable then reactivated 5 months after initiating ABA
17	RA	NA	93	< 10	39	ADA	18	–	63	90	33	RTX	No	No	Reactivation 7 months after detecting HBV DNA

Definitions: HBsAb < 10 mIU/mL: negative; HBV DNA < 10 IU/mL: undetectable; ALT < 40 IU/L: within normal limit

*ABA* abatacept, *ADA* adalimumab, *ALT* alanine transaminase, *AS* ankylosing spondylitis, *DMARD* disease-modifying anti-rheumatic drug, *ETA* etanercept, *GOL* golimumab, *ID* case code number, *LEF* leflunomide, *MTX* methotrexate, *NA* not available, *Pd* prednisolone, *RA* rheumatoid arthritis, *RTX* rituximab, *sAb* surface antibody, *sAg* surface antigen, *TCZ* tocilizumab

**Fig. 2:**
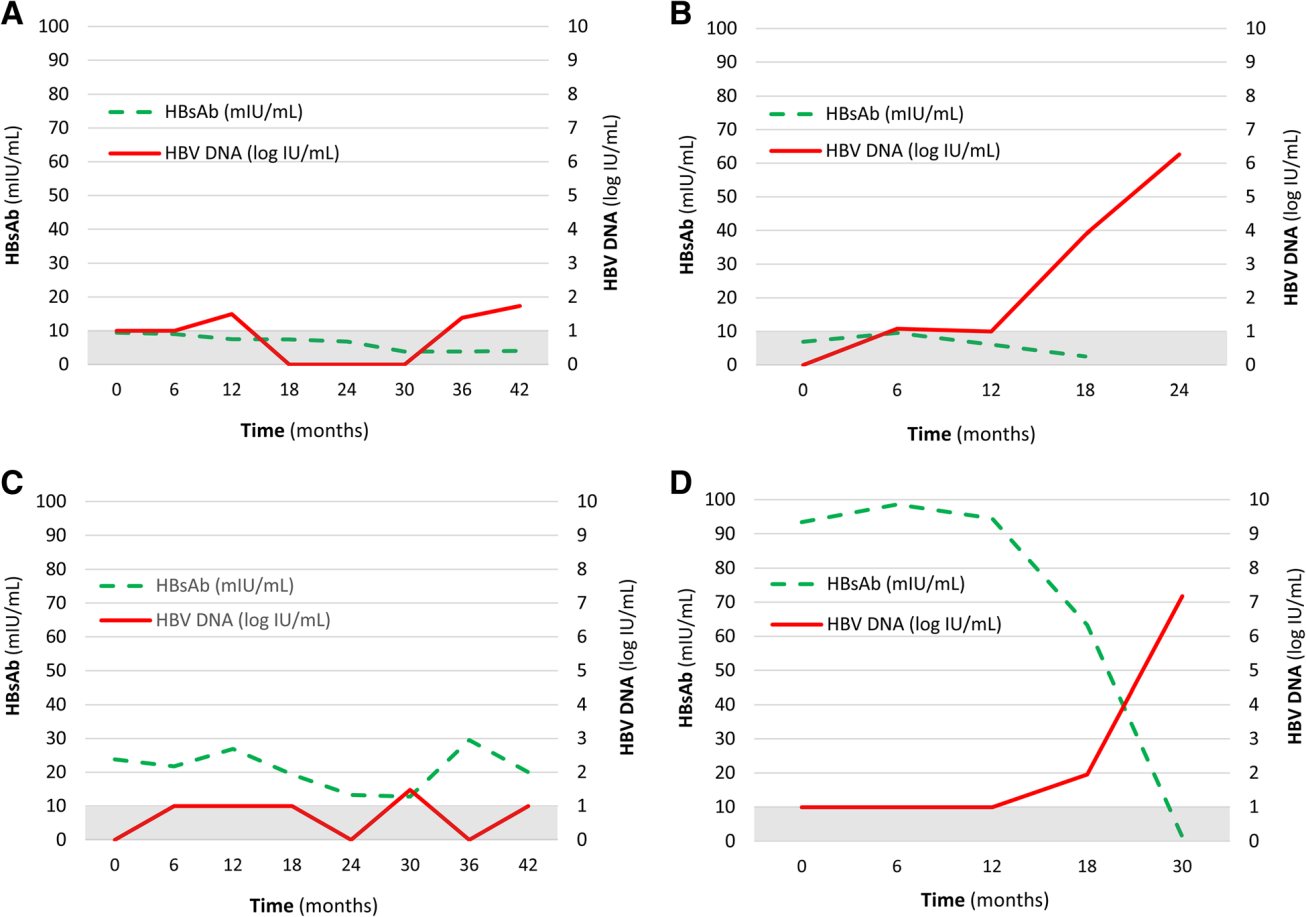
Different trends of hepatitis B virus surface antibody (HBsAb) titer associated with hepatitis B virus (HBV) DNA load. **a** Persistently HBsAb negative and HBV DNA detected periodically, without reactivation. **b** Persistently HBsAb negative, with HBV DNA detection followed by HBV reactivation. **c** Persistently low and HBV DNA detected periodically, without reactivation. **d** HBsAb titer changed from low to negative, followed by detectable HBV DNA, even HBV reactivation

Figure 2 shows the trends of HBsAb titer and associations with HBV DNA load, showing four patterns: a) persistently ‘negative’ during treatment and HBV DNA detected periodically, without reactivation (Table 2, cases 1–6 and 15); b) persistently ‘negative’ during treatment, with HBV DNA detection followed by reactivation (case 16); c) persistently ‘low’ during treatment and HBV DNA detected periodically, without reactivation (cases 8–14); and d) initially ‘low’ then becoming ‘negative’ during treatment, followed by HBV DNA elevation, and even HBV reactivation (cases 7 and 17).

## Discussion

We believe this to be the first reported investigation of dynamic changes in HBsAb titer and the associated risk of HBV reactivation in patients with HBsAg^−^/HBcAb^+^ serostatus receiving biologic therapy for rheumatic diseases. HBsAb titers changed during biologic therapy. Negative HBsAb serostatus was associated with HBV reactivation, whilst patients with low HBsAb titer had detectable HBV DNA without overt HBV reactivation. These findings suggest that the risk of HBV reactivation changes over time in HBsAg^−^/HBcAb^+^ patients receiving biologic therapy, and should be stratified according to the present, rather than baseline, HBsAb titer; serial HBsAb monitoring may enable earlier detection of HBV reactivation.

Low or negative baseline HBsAb titer is an important risk factor for HBV reactivation, whereas a high titer at baseline is protective [9, 10, 19]. However, following biologic therapy, several mechanisms may impair host antiviral defenses, resulting in HBsAb reduction and even loss [5]. HBsAb titer decreased among 21 patients after commencing anti-tumor necrosis factor (TNF) treatment, especially in those with a low baseline HBsAb [14]. In a larger observational study, HBsAb also declined among patients receiving non-anti-TNF biologic agents [15]. Although HBsAb titers in our follow-up cohort mostly remained little changed from baseline, some did change, typically showing a decline during biologic therapy. Such changes and the associated risk of HBV reactivation during biological therapy might be missed if HBsAb is only checked at baseline. Thus, the risk of HBV reactivation may be stratified according to changing HBsAb serostatus by continuous monitoring instead of using the baseline assessment only. First, HBsAb titer > 100 mIU/mL is protective. Second, transient HBV DNA viremia may be detected sporadically in patients with HBsAb 10–100 mIU/mL, which may be benign while the HBsAb titer stays low. Finally, patients with negative HBsAb serostatus (whether initial or seroconverted) could have an increased risk for HBV reactivation.

Although serum HBV DNA may be undetectable in HBsAg^−^/HBcAb^+^ patients, the virus persists for life in hepatocyte nuclei as covalently closed circular DNA [20]. After immunosuppressive therapy and HBsAb reduction, HBV DNA may become detectable in sera. Clinical studies have reported patients with detectable HBV DNA during biological or immunosuppressive therapy that later resolved spontaneously, without antiviral therapy; this transient self-limiting viremia may not lead to HBV DNA replication or overt hepatitis [3, 21, 22]. Congruently, 17 of our patients with negative or low HBsAb titer manifested detectable HBV DNA, most with no HBV reactivation, without preemptive antiviral therapy. Serial HBV DNA assays showed levels that fluctuated or spontaneously became undetectable during biological therapy without hepatitis. Furthermore, all patients with transient HBV DNA viremia without apparent reactivation had low HBsAb serostatus, while two with HBV reactivation had negative HBsAb serostatus, which indicates that current HBsAb titer is more important than baseline HBsAb in determining the risk of HBV reactivation. However, the clinical significance and decisions about whether to start antiviral treatment for patients with low HBsAb titer and transient HBV DNA viremia require further elucidation.

This study had limitations. We could not evaluate the risk of HBV reactivation associated with biologic therapy for several reasons. First, the use of biologic therapy varied over time in many cases. Second, combinations of other immunosuppressive agents differed in various rheumatic diseases, which prevented accurate estimation of the long-term cumulative effects of biologic and immunosuppressive agents. Third, there is currently no standard definition of HBV reactivation in HBsAg^−^/HBcAb^+^ patients; therefore, the incidence rate of HBV reactivation should be interpreted cautiously. Furthermore, although we observed that the infusion of rituximab markedly reduced the titer of HBsAb in some patients, the small number of cases is not sufficient to reach solid conclusions regarding the influence of rituximab on HBsAb decreases or HBV reactivation. Besides, small case numbers also limit multivariate analysis to confirm risk factors associated with HBV reactivation. Larger sample sizes to explore the features associated with HBV reactivation are required.

## Conclusions

HBsAb titer in patients with HBsAg^−^/HBcAb^+^ serostatus changes during biologic therapy and their risk of HBV reactivation varies over time according to changing HBsAb titer. High HBsAb titer wards off detectable HBV DNA and HBV reactivation. Patients with low HBsAb titer may present transient HBV DNA viremia with a benign course, whereas negative HBsAb serostatus is associated with an increased risk of HBV reactivation.

## Additional file

Additional file 1: **Figure S1.** illustrated the titers of HBsAb from baseline to the end of follow-up of all patients. (TIF 434 kb)

## Abbreviations

CI: Confidence interval
HBcAb: Hepatitis B virus core antibody
HBsAb: Hepatitis B virus surface antibody
HBsAg: Hepatitis B virus surface antigen
HBV: Hepatitis B virus
PY: Person-years
TNF: Tumor necrosis factor
