# Supplementary figures and images for: Changes in hepatitis B virus surface antibody titer and risk of hepatitis B reactivation in HBsAg-negative/HBcAb-positive patients undergoing biologic therapy for rheumatic diseases: a prospective cohort study
Source: Arthritis Res Ther. 2018 Nov 1;20:246. doi: 10.1186/s13075-018-1748-z (PMC6235201; doi:10.1186/s13075-018-1748-z)

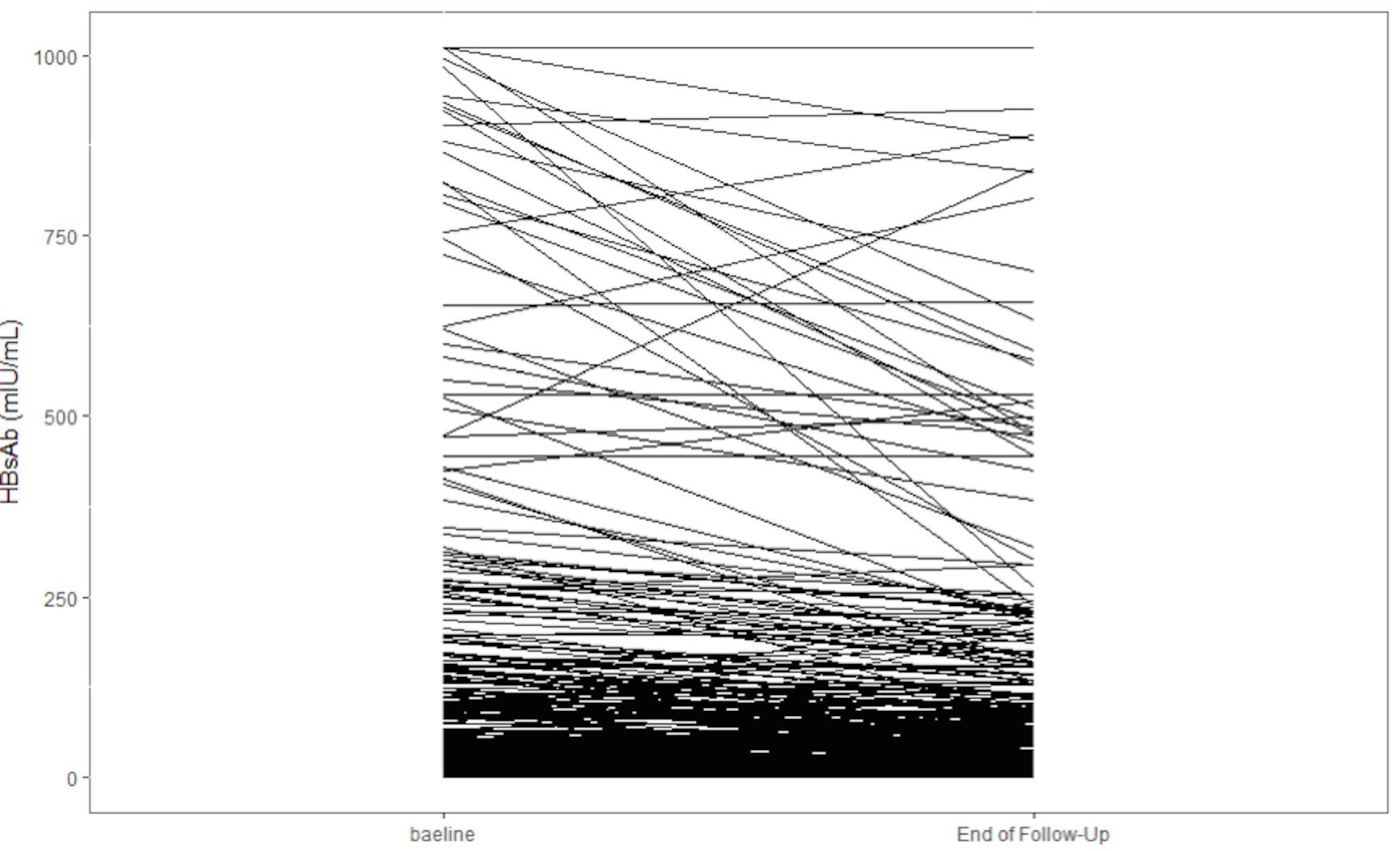

Supplement: Supplementary file 1 — Figure S1. illustrated the titers of HBsAb from baseline to the end of follow-up of all patients. (TIF 434 kb) [file 13075_2018_1748_MOESM1_ESM.tif]
